# Interaction effects of glycine equivalent and standardized ileal digestible threonine in low protein diets for broiler grower chickens

**DOI:** 10.5713/ab.23.0307

**Published:** 2024-02-22

**Authors:** Paschal Chukwudi Aguihe, Amanda Barroso Castelani, Camilo Ivan Ospina-Rojas, Eustace Ayemere Iyayi, Paulo Cesar Pozza, Alice Eiko Murakami

**Affiliations:** 1 Department of Animal Production and Heath Technology, Federal College of Wildlife Management, P.M.B 268, New Bussa 912106, Nigeria; 2 Departamento de Zootecnia, Universidade Estadual de Maringá, Maringá, Paraná, 87020-900, Brazil; 3 CJ Corporation, Monsões, São Paulo 04571-010, Brazil; 4 Department of Animal Science, University of Ibadan, Ibadan 200005, Nigeria

**Keywords:** Broiler, Glycine Equivalent, Muscle Creatine, Performance, Serum Biochemistry, Threonine

## Abstract

**Objective:**

This study aims to investigate the interactive effect of a glycine equivalent (Gly_equi_) and standardized ileal digestible threonine (SID Thr) levels in low crude protein diets on performance, blood biochemistry, pectoral muscular creatine content and oxidative stability of meat in broiler chickens from 21 to 42 days.

**Methods:**

A total of 1,500, twenty-one-day-old Cobb-Vantress male broiler chickens were distributed in a completely randomized 5×3 factorial arrangement of Gly_equi_×SID Thr with five replicates of 20 birds each. Fifteen dietary treatments of 16.5% CP were formulated to contain five levels of total Gly_equi_ (1.16%, 1.26%, 1.36%, 1.46%, and 1.56%) and three levels of SID Thr (0.58%; 0.68% and 0.78%).

**Results:**

Interaction effects (p<0.05) of Gly_equi_ and SID Thr levels were observed for weight gain, carcass yield, pectoral muscular creatine content and serum uric acid. Higher levels of Gly_equi_ increased (p = 0.040) weight gain in 0.58% and 0.68% SID Thr diets compare to the 0.78% SID Thr diet. The SID Thr level at 0.68% improved (p = 0.040) feed conversion compared to other SID Thr diets. Levels of Gly_equi_ equal to or above 1.26% in diets with 0.78% SID Thr resulted in birds with higher (p = 0.033) pectoral muscular creatine content. The breast meat yield observed in the 0.68% SID Thr diet was higher (p = 0.05) compared to the 0.58% SID Thr diet. There was a quadratic effect of Gly_equi_ levels for pectoral pectoral muscular creatine content (p = 0.008), breast meat yield (p = 0.030), and serum total protein concentrations (p = 0.040), and the optimal levels were estimated to be 1.47%, 1.35%, and 1.40% Gly_equi_, respectively. The lowest (p = 0.050) concentration of malondialdehyde in the breast meat was found in 0.68% SID Thr diets at 1.36% Gly_equi_.

**Conclusion:**

The minimum dietary level of Gly_equi_ needed to improve performance in low crude protein diets is 1.26% with adequate SID Thr levels for broiler chickens.

## INTRODUCTION

Reducing the crude protein (CP) content in diets for broilers has created a mechanism capable of decreasing feed costs and nitrogen emissions from the production of meat products [1,2]. However, low-protein diets tend to impair performance by decreasing weight gain, worsening feed conversion, and increasing carcass fat content [3]. The protein requirement in the diet of birds involves both the need for essential amino acids (AAs) and the supply of non-essential AAs and nitrogen. Meeting the nutritional requirements of essential AAs makes it possible to carefully reduce the CP in the diet without harming the performance of the birds [4]. The concern with meeting only the needs of essential AAs increase the chance of a non-essential AAs becoming limiting in the diet, due to the amount of precursors for a given AA being insufficient or due to the delay of metabolic processes [5]. Dietary supplementation of glycine (Gly), which is a non-essential AA, was shown to be able to promote improvement in the productive parameters of broilers during the starter and grower phases [4,6,7]. Even with the ability of birds to synthesize Gly endogenously, this synthesis may not be metabolically efficient to meet the AA requirements of broilers in the initial phase [8]. Thus, the diet must be formulated to supply at least 40% of the total Gly requirement to enable the growing birds to meet the necessary metabolic demand for protein deposition and production of biomolecules, such as uric acid (UA), creatine, glutathione and collagen [9]. Since Gly can be reversibly metabolized to serine (Ser) and, on a molar basis, Ser plays the same functions as Gly in chicken, it becomes necessary that the requirements of these AAs be considered jointly as Gly equivalents (Gly_equi_) in diet formulations [4].

Moreover, threonine (Thr) serves as a possible metabolic precursor of Gly and occurs as an active process through a series of enzymatic reactions [9], which is potentially necessary in fulfilling the Gly requirement for poultry [4,10,11]. When Thr is degraded by the Thr aldolase enzyme, Thr generates Gly and acetaldehyde as by-products or via Thr dehydrogenase with 2-amino-3-ketobutyrate as an intermediate step generating acetyl-CoA, which can be directly converted into Gly [12]. Thr being considered as the third limiting AA in corn-soybean meal-based diets provided to broilers, in addition to playing a fundamental role in the synthesis of proteins and mucin, also allows the lowest concentration of Gly_equi_ to be contained in the diets [13,14]. Studies have shown that diets with marginal Gly_equi_ levels, which is commonly observed in low-CP diets formulated with plant-based ingredients, can be viable by having enough Thr for broilers [10,13,15]. If the premise that increasing dietary Thr in low-protein diets increases performance is true, this suggests that Thr concentrations may be acting equivalently with Gly, demonstrating that these AAs may be crucial for diets formulated with reduced CP content. Thus, the present study was conducted to evaluate the Gly_equi_ requirements of broilers from 21 to 42 days of age, by offering reduced CP diets with varying standardized ileal digestible threonine (SID Thr) levels.

## MATERIALS AND METHODS

### Animals, facilities, and management

All procedures adopted were approved by the Ethics Committee on the Use of Animals (CEUA) of Maringá State University (CEUA/PROTOCOL-7501230419/2019). The experiment was conducted in the poultry section of the Experimental Farm of Iguatemi (FEI), which belongs to the State University of Maringá (UEM), using one thousand five hundred 21-day-old male broilers of the commercial strain Cobb-Vantress. The birds were housed in a conventional air-conditioned shed, with negative pressure ventilation and an evaporative plate, in 1.0×2.0-meter boxes, using new rice husk bedding. Water and feed were provided *ad libitum* throughout the experimental period through nipple drinkers and tube feeders, respectively. The lighting program used was continuous light 24 hours a day.

### Experimental design and diets

The broilers (average initial body weights of 1,083±5.1; p = 0.82) were distributed in a completely randomized design of 5×3 factorial arrangement of treatments (Gly_equi_×SID Thr), with five replications of 20 birds per experimental pen. The treatments consisted of five levels of Gly_equi_ (1.16%, 1.26%, 1.36%, 1.46%, and 1.56%) and three levels of SID Thr (0.58%, 0.68%, and 0.78%, corresponding to 85%, 100%, and 115% of the recommended SID Thr by Rostagno et al [16], respectively). The birds received a conventional diet in the initial phase (1 to 21 days of age) and the experimental diets in the growth phase, from 21 to 42 days of age. A basal diet with 16.5% CP was formulated according to the chemical composition values of the feed and the nutritional recommendations for male broilers of average performance as suggested by Rostagno et al [16], except for the concentrations of Gly_equi_ and SID Thr (Table 1). The other experimental diets containing different concentrations of dietary Gly_equi_ and SID Thr were obtained by supplementing L-Gly and L-Thr in the basal diet mixture at the expense of the inert (Kaolin; Minasilicio Gma Mineradora Ltd, Jequitibá, MG, Brazil). The feed formulation based on dietary Gly_equi_ (Gly_equi_ (g/kg) = Gly+0.7143 ×Ser) was necessary since on a molar basis, the nutritional interrelation of Gly and Ser makes it necessary to evaluate together the dietary needs of these AAs [4].

### Productive performance

Birds and the experimental diets were weighed at 21, 28, 35, and 42 days of age for performance evaluation (weight gain, feed intake, and feed conversion ratio [feed:gain]). Average daily gain (ADG) was calculated using initial and final weight data on a week. Feed intake on weekly basis was measured based on feed disappearance in each pen. Thereafter, mortality-corrected average daily feed intake (ADFI) and feed:gain of each pen were calculated in each respective week throughout the duration of the experiment as described by Hofmann et al [17].

### Carcass and cuts yield

At 42 days of age, one bird from each experimental unit was selected according to the mean weight (±5%) of the repetition, and fasted for 6 hours, then slaughtered by stunning with electric shock and subsequent bleeding. After complete immobilization of the birds, carcasses were scalded at 60°C for 30 seconds and then defeathered using a rotary plucker according to the procedure of Elahi et al [18]. The carcass yield was determined by taking the weight of the eviscerated carcass, excluding feet, head, neck, and abdominal fat in relation to the live weight of the birds that were individually weighed before slaughter. Also, the yield of the whole breast and legs (thigh + drumstick) was calculated in relation to the weight of the eviscerated carcass. The abdominal fat present around the cloaca, cloacal bursa, gizzard, proventriculus and adjacent abdominal muscles were removed, weighed and its absolute weight expressed as a percentage of live weight of the bird.

### Serum biochemistry

At 42 days of age, blood samples (±2 mL/bird) were collected into sterile vacuum tubes from the jugular vein of two birds per experimental unit, selected based on the mean weight of the cage (±5%). The samples were kept on ice and centrifuged at 3,000 RPM for 10 mins (Excelsa, Baby II 206 R, São Paulo, Brazil), the serum samples being stored at −80°C until the analysis was performed. The serum samples were thawed at 4°C and the serum concentrations of triglycerides, UA, total proteins, albumin, creatinine and glucose were analyzed using an enzymatic-colorimetric process (Gold Analise Diagnóstica Ltd, Belo Horizonte, MG, Brazil) with the aid of a spectrophotometer (model BIOPLUS 2000; Bioplus Ltd, São Paulo, Brazil), according to procedures described by Tietz [19]. Serum ammonia was determined as described by Ishihara et al [20], a procedure in which ammonia reacts with 2-ketoglutarate and nicotinamide adenine dinucleotide (NADH) in a reaction catalyzed by the enzyme glutamate dehydrogenase, resulting in the oxidation of NADH to NAD^+^.

### Pectoral muscular creatine content and relative organ weight

At 42 days of age, one bird was selected from each experimental pen and slaughtered. Breast muscle, liver and pancreas were removed and weighed on a precision scale (Gehaka BK400; Gehaka Ltd, São Paulo,, Brazil) to obtain the relative weight (%) in relation to live weight of the bird. Thereafter, samples of the pectoral muscle of the selected birds were collected, weighed, and ground in a mixer type mill (Maxmac ZJB750; Maxmac Ltd, São Paulo, Brazil). The creatine concentration in the pectoral muscle was determined following the methodology of Chamruspollert et al [21].

### Meat oxidative stability

Oxidative stability was evaluated using the thiobarbituric acid reactive substances (TBARS) and equivalent oxidation in malonaldehyde (MDA) methodology as described by Sørensen and Jørgensen [22]. A completely randomized design was used, in a 3×3 factorial arrangement of treatments, with three levels of total Gly_equi_ (1.16; 1.36% and 1.56%) and three SID Thr levels (0.58; 0.68% and 0.78%) using five replications per treatment. Breast meat from one bird per repetition was stored in a freezer at −18°C and a sample of 50 g per replication was taken. The samples were ground in a food processor (Maxmac ZJB750; Maxmac, Brazil) and 5 g was reserved, which was placed in a glass tube. The TCA solution of 15 mL (7.5% trichloroacetic acid), gallic acid (0.1%), and ethylenediaminetetraacetic acid (0.1%) were added. Subsequently, with the aid of a Turax, the sample was homogenized for one min and placed to filter through filter paper (12.5 mm). A 1.5 mL aliquot of the filtered solution was added to 1.5 mL of 2-thiobarbituric acid (TBA) (0.02 M) in a test tube and heated in a water bath at 100°C for 40 mins. The tubes were cooled and centrifuged for 10 mins at 3,000 rpm. Then, the absorbance reading was performed in a spectrophotometer at a wavelength of 540 nm. To perform the calculations, the MDA standard curve was used, and the results were expressed in mg of MDA/kg of meat.

### Statistical analysis

The experiment adopted a completely randomized design of 5×3 factorial arrangement of treatments. The data obtained were subjected to 2-factor analysis of variance using the general linear model procedure of the SAS University Edition statistical program by the following model:


$${\text{Y}}_{\text{ijk}}=\mu +{\text{G}}_{\text{i}}+{\text{T}}_{\text{j}}+{\text{GM}}_{\text{ij}}+{\text{e}}_{\text{ijk}}$$

Where Y_ijk_ is the response variable, μ is the common mean; G_i_ is the effect of the ith Gly_equi_; T_j_ is the effect of the jth SID Thr; GT_ij_ is the effect of the interaction of the ith Gly_equi_ with the jth SID Thr; and e_ijk_ is the error term. The replicate pen was used as an experimental unit for analyzing the data. When the analysis of variance indicated significant treatment effects, means were separated using Tukey’s multiple range tests by LSMEANS procedure of SAS program. Statistical differences were considered to be significant at p<0.05. A polynomial regression model was applied and where quadratic responses (p<0.05) were detected, the optimal Gly_equi_ level was calculated by taking the first derivative of the quadratic equation.

## RESULTS

### Growth performance

At 42 days of age, the interaction effect of Gly_equi_ and SID Thr levels was significant (p = 0.04) for weight gain but feed intake and feed:gain were not affected (Table 2). Results showed that higher (p = 0.040) weight gain was recorded for diets containing 0.58% and 0.68% SID Thr with increasing levels of Gly_equi_. Thus, birds fed with the adequate (0.68%) SID Thr diets with more than 1.26% Gly_equi_ and deficient (0.58%) SID Thr diets containing Gly_equi_ levels equal to or greater than 1.36% showed higher (p = 0.040) weight gain. Among diets with 0.78% SID Thr, birds on 1.16% Gly_equi_ had increased (p = 0.040) weight gain compared to those on 1.26 to 1.56% Gly_equi_. The main effect of increasing dietary levels of Gly_equi_ did not have any influence on the weight gain per feed intake and feed:gain of the birds. Feed:gain was influenced (p = 0.03) by different dietary SID Thr concentrations and birds that received the diet with 0.68% SID Thr showed lower (p = 0.03) feed:gain compared to those fed with the 0.78% SID Thr diet.

### Muscle creatine, relative weights of liver and pancreas

There was an interaction effect (p = 0.033) between the levels of total Gly_equi_ and SID Thr for creatine content in the pectoral muscles but no effect was recorded for the relative weight of the liver and pancreas at 42 days (Table 3). The results of the present study revealed that broilers fed with 0.58% and 0.68% SID Thr diets showed higher (p = 0.033) pectoral muscular creatine values at Gly_equi_ concentrations of 1.36% and 1.46% respectively. However, pectoral muscular creatine levels were lower (p = 0.033) in birds fed with 0.58% Thr diets at 1.16% and 1.56% Gly_equi_ levels and in the 0.68% Thr dietary group at 1.26% Gly_equi_. In diets with 0.78% SID Thr, pectoral muscular creatine content was lower (p = 0.033) in diets containing 1.26% Gly_equi_ compared to other Gly_equi_ levels. Moreover, results showed that a quadratic effect (p = 0.008) of increasing levels of total Gly_equi_ was observed for pectoral muscular creatine content, with an estimated optimal level of 1.47% Gly_equi_. Also, the pectoral muscular creatine concentration in the birds fed with diets containing 0.68% and 0.78% SID Thr were higher (p = 0.048) than those in the 0.58% SID Thr group.

### Carcass characteristics

The effects of increasing levels of Gly_equi_ and SID Thr in the diets of growing broiler chickens on carcass characteristics are presented in Table 4. There was an interaction (p = 0.002) between dietary concentrations of Gly_equi_ and SID Thr for carcass yield. In diets with 0.58% and 0.68% SID Thr concentrations, providing respective Glye_qui_ levels at 1.56% and 1.36%, resulted in higher (p = 0.002) carcass yield than 1.16% Gly_equi_. Birds given a 0.78% SID Thr diet in combination with 1.16% and 1.46% Gly_equi_ had an increased (p = 0.002) carcass yield compared to those given the same diet in combination with 1.56% Gly_equi_. The main effect (p<0.05) of the Gly_equi_ levels were observed for relative weight of breast and abdominal fat. A quadratic effect (p = 0.03) of Gly_equi_ levels was obtained for breast yield, with an optimal level estimated at 1.35% Gly_equi_. On the other hand, increasing levels of Gly_equi_ resulted in a decreased linear effect (p = 0.01) on the relative weight of abdominal fat. Moreover, the different dietary levels of SID Thr did not influence carcass attributes of the birds except for breast yield. Feeding birds with a diet containing 0.68% SID Thr showed a tendency for a higher (p = 0.05) breast yield than that of the birds with fed 0.58% SID Thr.

### Blood biochemistry

At 42 days, all the blood variables evaluated in the present study showed no interaction between dietary levels of Gly_equi_ and SID Thr, except for UA (p = 0.002) (Table 5). In birds fed with diets containing 0.58% SID Thr, a decrease (p = 0.002) in serum uric acid (SUA) concentrations was observed at 1.36%, 1.46%, and 1.56% Gly_equi_ compared to 1.16% and 1.26% Gly_equi_. Additionally, birds fed with diets containing a 0.68% SID Thr concentration recorded lower (p = 0.002) SUA at 1.26% and 1.46% Gly_equi_ compared to those containing 1.56% Gly_equi_ which had higher (p<0.05) SUA values. In diets with 0.78% SID Thr concentration, SUA concentration of the birds were similar among the different Gly_equi_ levels. The main effect of the dietary levels of Gly_equi_ did not affect all the blood variables evaluated, only serum total proteins, which showed a quadratic effect (p<0.05) with an estimated optimal level of 1.40% Gly_equi_. An increase in the concentration of SID Thr from 0.58% to 0.78% in the diet of grower broilers showed no significant difference in any of the blood parameters measured.

### Meat oxidative stability

The interaction between Gly_equi_ and SID Thr levels for the oxidative stability of breast meat was not significant, although it showed a tendency for decreased (p = 0.05) TBARS concentrations across the various SID Thr diets at increasing levels of Gly_equi_ (Table 6). The TBARS values were observed to decrease (p = 0.03) linearly as the levels of Gly_equi_ increased from 1.16% to 1.56%. Birds fed with 1.16% and 1.36% Gly_equi_ diets showed similar TBARS values but higher (p = 0.03) than the group fed diet with 1.56% Gly_equi_. No significant effect of increasing the dietary SID Thr concentration from 0.58% to 0.78% was recorded for meat oxidative stability in the growing broiler chickens.

## DISCUSSION

Lowering the dietary CP content offers several significant advantages for sustainable chicken meat production, including reduced dietary costs, lower emissions of nitrogen and ammonia, improved litter quality, and enhanced bird welfare due to less indigestible protein entering the hindgut to support the growth of potential pathogens [2,23,24]. A substantially reduced-CP diet for broiler chickens raised during the growing period contains between 2% and 3% less CP compared to a conventional diet [10,25]. Thus, the present study examined a CP concentration of 16.5%, which is lower than the Cobb-Vantress [26] recommended requirements for corn-soybean based diets. Since low-CP diets are gaining popularity in poultry nutrition, it is essential to meet the Gly_equi_ and threonine requirements of broiler chickens for optimal growth performance because these AAs are considered essential in low-CP corn-soybean-meal-based diets [5,13,27]. The present study investigated the interactive effect of a Gly_equi_ and SID Thr levels in low CP diets on the performance of growing broilers (22 to 42 d of age). The results obtained for weight gain revealed that in low-protein diets, Thr supplementation at levels lower than and/or equal to recommended SID Thr could increase the Gly_equi_ requirements. However, supplementing Thr to provide excess dietary SID Thr levels reduces the requirement of Gly_equi_, thus confirming the result of previous studies [9,10,13]. This shows that the decline in weight gain of birds fed with low-protein diets could be prevented when excess SID Thr is provided with marginal Gly_equi_ levels. Chrystal et al [28] observed that, when there is deficient dietary Gly_equi_ levels, Thr reduces feed consumption, causing losses in the productive response of birds. However, some studies have shown that, excess Thr supplementation reduces the need for Gly_equi_, since the enzymes Thr aldolase and Thr dehydrogenase are able to degrade excess Thr into Gly [8,13]. Consequently, more energy (9 ATP/mol) needed in the synthesis of Gly is conserved, hence making an increased amount of energy available for optimal growth [10]. Interactive effects between total Gly_equi_ and SID Thr intake have been frequently reported in the literature [14,15,28]. Ospina-Rojas et al [10] providing diets with 0.84% to 0.92% Thr and 1.44% to 1.76% Gly_equi_ observed an interaction between Gly levels and Thr levels feed:gain, but not in weight gain in broilers aged 21 to 35 days. On the other hand, Corzo et al [13], working with grower broilers fed with diets containing 0.72% to 0.81% Thr and 1.64% to 1.76% Gly_equi_, obtained an interaction for weight gain, but not for feed:gain. According to Bernardino et al [29], Gly supplementation decreases the activity of enzymes that catabolize Thr, thus saving Thr from being degraded to Gly. Moreover, Ospina-Rojas et al [10] demonstrated that Gly supplementation would be necessary to maintain dietary Thr levels rather than reducing it, because the use of Thr for physiological roles such as production of gastrointestinal mucin, among other functions, would be reduced via Gly addition, thus resulting in improved growth rates in broilers. Therefore, increased Thr supplementation is unnecessary in diets containing adequate or high Gly_equi_ concentrations; because excess Thr compromises the productive performance of birds, increasing the elimination of UA, as this process requires high consumption of metabolic energy [10]. Also, our results are in accordance with earlier reports that showed Thr supplementation at levels higher than its recommendation could serve as a source of dietary Gly_equi_ with marginal AA concentrations, promoting the use of low-protein diets for broilers without any negative effects on growth performance [10,15]. Thus, this outcome shows evidence to support the idea that the needs for Gly_equi_ are reduced when dietary SID Thr is provided in excess of its requirement. The feed:gain in this present study tended to be lower when adequate SID Thr concentration in the diet was offered to the birds compared to when SID Thr levels exceeded its requirement. This was in line with the results of previous studies, which have shown that the feed:gain of birds improved when they received the appropriate concentration of the Thr amino acid [8,10,30]. The feed:gain of birds that received the adequate level (0.68%) of SID Thr may have improved due to the stabilization and maintenance function of the intestinal tract performed by Thr, favoring the digestion of nutrients [10,31]. These same authors point out that adequate levels of Thr allow the digestive system to benefit from increased amylase secretion and mucin production, improving the digestibility of nutrients present in the diet.

An increase in the requirement of Gly_equi_ resulted in increased pectoral muscular creatinein of birds fed with varying dietary levels of SID Thr with reduced CP concentration. Since the need for Gly_equi_ in the synthesis of creatine is considered essential at the starting period of the broiler’s growth, increasing levels of Gly_equi_ resulted in a positive response with respect to pectoral muscular creatine during grower stage (22 to 42 d) when low-protein plant-based diets are provided. The present study suggests that Thr supplementation to provide dietary SID Thr at levels lower or greater than the required (0.68%) showed an increment in the Gly_equi_ requirement for muscle creatine, thus confirming the report of Ospina-Rojas et al [8] and Aguihe et al [15]. Creatine, in addition to having considerable importance in increasing growth performance, can also be considered an important source of energy for muscle tissue, since it allows the immediate resynthesis of ATP [32]. Furthermore, when the amount of pectoral muscular creatineis increased, there is a better use of nutrients for muscle growth in this tissue [33]. However, plant-based diets do not provide a sufficient amount of creatine as required by birds, as there is inadequate dietary creatine supplied via plant ingredients. Therefore, to supply this deficit, greater amounts of Gly are required to synthesize creatine [34]. Therefore, increasing total Gly_equi_ up to 1.56% in the diet through Gly supplementation may be favorable as a source of creatine, thus, promoting improved utilization of nutrients and energy for proper muscle growth in this tissue [33]. SID Thr levels influenced the pectoral muscular creatine content, which was higher in broilers that received adequate (0.68%) and excessive (0.78%) SID Thr diets compared to those offered deficient SID Thr (0.58%). This result suggests that increasing dietary SID Thr at or above recommended level in broilers fed with low-protein diets is essential for promoting the synthesis of creatine in muscle tissues. One possible explanation for the positive response to increasing Thr levels on pectoral muscular creatine is that it acts as a readily available precursor to Gly, thus invariably increasing the amount of Gly_equi_ needed for the creatine synthesis pathway [8]. This observation is supported by a previous report that demonstrated that the catabolism of Thr yields Gly to support metabolic processes such as the conversion of guanidino acetic acid to creatine [35]. Therefore, the synthesis of creatine depends on increased availability of Gly_equi_ for methylation of guanidino acetic acid, which is the only metabolic intermediary product and precursor of creatine in muscles [36]. Creatine levels are good indicators of protein and AA metabolism, which stimulates protein synthesis in muscles and are involved in energy metabolism and storage [37].

In the current experiment, carcass yield was influenced by the interaction of Gly_equi_ and SID Thr levels in the diet. The results obtained for carcass yield suggest that dietary Thr had a compensatory effect under the lowest level of Gly_equi_ in the diet of birds aged 22 to 42 d. This implies that since the enzymes Thr aldolase and Thr dehydrogenase can catabolize excess Thr into Gly, Thr would be able to decrease Gly requirements. However, in situations of marginal levels of Gly in a diet based on low protein plant-based products, Thr can be considered a relevant source of Gly [10,14]. Similar results were found by Corzo et al [13] for which lower Gly_equi_ levels resulted in lower carcass yield values that only improved when dietary Thr was increased. Still, Baker et al [9] mentioned that when Thr was shown to be in moderate excess, the Gly-sparing effect of dietary Thr was found. The influence of Gly_equi_ levels on breast yield may be associated with the increase in creatine content in the pectoral muscles in response to Gly supplementation, as creatine is considered an important energy source in animal feed [32]. Unlike by-products of animal origin, ingredients of plant origin do not meet the nutritional requirement for the performance of birds, thus increasing the needs of Gly, an amino acid used for the endogenous synthesis of creatine [33]. Dietary Gly_equi_ levels linearly decreased (p<0.05) the abdominal fat content. A previous study illustrated the challenge of providing diets with low CP, as these compromise feed conversion and led to greater fat deposition [38]. According to Ospina-Rojas et al [10], digestion of dietary fat is favored by Gly supplementation, considering that this AA is a component of bile salts, constituting about 90% of the total AA present in the bile.

The result of the present study reveal that higher Gly_equi_ is necessary in low-protein diets with a lower concentration of SID Thr to achieve a lower SUA, while Gly_equi_ requirements decreased in diets with adequate SID Thr for growing broilers, which may be a reflection of greater efficiency in the use of AAs. The synthesis of UA as the final product of AA catabolism in chickens, is a Glyequi-dissipating process to eliminate excess nitrogen [13]. SUA is used as an influential parameter to reflect the extent of amino acid utilization in broiler chickens. In SID Thr-deficient low-protein diets, the linear decrease in SUA concentration with increasing Gly_equi_ levels is an indication of the reduced rate of AA oxidation to spare more protein for improved nitrogen utilization [39,40]. The increase in SUA concentration in diets with 0.78% Thr in response to Gly_equi_ supplementation may be due to an AA imbalance. Dietary AAs when in excess, are deaminated into carbon and ammonia skeleton which, due to their high toxicity to animal tissues, are eliminated as UA by birds [38]. In poultry, the premise is that UA is the main product of nitrogen excretion; and for each UA molecule formed, a Gly molecule is lost, an indication of a higher metabolic requirement for Gly in broilers [4]. This could explain the importance of supplementing diets low in CP with adequate levels of essential and non-essential AAs so that the rate of AA oxidation decreases to save more protein for improved performance in broiler chickens [41]. Increasing dietary Gly_equi_ levels showed a quadratic response on total protein at an optimum level of 1.40% in the current study. This is an indication that the adequate levels of Gly_equi_ in dietare necessary to optimize cellular protein synthesis, since total proteins can be used as markers of protein nutritional status [1].

The findings of the present study show that increased TBARS concentrations associated with higher lipid peroxidation in birds fed with imbalance SID Thr diets were mitigated by the increased supply of dietary Gly_equi_ provided through Gly supplementation. The main function of Gly is to favor the excretion of excess nitrogen by donating carbon and nitrogen to the synthesis of UA, this AA is also involved in the process of synthesizing glutathione, which plays an antioxidant role in the body of animals [42]. Glutathione is an important bio-compound that neutralizes and protect cells against the damaging effects of free radicals by enhancing lipid peroxidation, a process leading to the oxidation of polyunsaturated fatty acids to lipid peroxides [42]. The existence of glutathionine as an antioxidant within cells hinders the generation of free radicals and advancement of peroxidation [43,44]. This could explain the observed positive influence of higher dietary total Gly_equi_ levels on lipid oxidation by preventing intracellular degradation of glutathionine via elevated glutathione peroxidase and glutathione reductase activity [45]. These enzymes convert radicals and peroxides in innocuous reduced forms, often with the concomitant oxidation of reduced glutathione to its oxidized form [43,45]. The current study corroborates the findings of Gao et al [45] and Deng et al [46] who demonstrated that Gly supplementation led to a significant increase in glutathione levels and upregulation of antioxidant-related genes, including glutathione peroxidase, catalase, and superoxide dismutase; as well as the decrease in lipid peroxidation caused by reactive oxygen species and a reduction in MDA levels. This positive effect was attributed to the stimulation of lipid metabolism via increased functionality of mitochondria, lipid droplets, and factors involved in lipid metabolism, such as peroxisome proliferator-activated receptors, sterol regulatory element-binding factor, and ATP [46]. Thus, this present result demonstrates the potential efficacy of sufficient dietary Gly_equi_ levels in protecting tissues from peroxidative damage.

## CONCLUSION

The Gly_equi_ needed for improved performance in low protein diets with adequate levels of SID Thr (0.68%) for broilers (21 to 42 days old) is 1.26% to 1.36% with marginal levels of SID Thr. For deficient (0.58%) and excessive (0.78%) SID Thr diets, increasing Gly_equi_ levels is necessary to enhance pectoral muscular creatine, carcass yield, meat oxidative stability and reduced SUA. Moreover, the optimal level of Gly_equi_ in low-protein diets are estimated at 1.47%, 1.35% and 1.40% for pectoral muscular creatine, breast yield and total protein, respectively for grower broilers.

## Figures and Tables

**Table 1:** Percentage and calculated ingredient composition of the basal diet for broilers in the growth phase (21 to 42 days of age)

Items	Composition
Ingredients (%)	
Corn, 8.8% CP	71.10
Soybean meal, 45% CP	20.94
Soybean oil	2.10
Dicalcium phosphate	1.20
Limestone	0.80
Salt	0.45
Min–Vit Supl.^1)^	0.40
Kaolin^2)^	1.50
DL-Met 99%	0.33
L-Lys HCL 78.5%	0.50
L-Thr 98%	0.07
L-Val 98%	0.18
L-Ile 98%	0.15
L-Arg 99%	0.24
L-Trp 98%	0.04
Calculated composition	
Crude protein (%)	16.5 (16.8)^3)^
AME (kcal/kg)	3.125
Calcium (%)	0.69
Chloride (%)	0.32
Available phosphorus (%)	0.32
Potassium (%)	0.58
Sodium (%)	0.20
Lys (%)	1.12 (1.14)
Met+Cys (%)	0.82 (0.80)
Thr (%)	0.66 (0.67)
Val (%)	0.90 (0.94)
Arg (%)	1.18 (1.24)
Ile (%)	0.77 (0.81)
Gly (%)	0.61 (0.68)
Ser (%)	0.77 (0.80)
Gly_equi_ (%)	1.16 (1.19)
SID Lys (%)	1.04
SID Met+Cys (%)	0.76
SID Thr (%)	0.58
SID Val (%)	0.82

CP, crude protein; AME, apparent metabolizable energy; Gly_equi_, glycine equivalent; SID, standardized ileal digestible.

1) Vitamin and mineral supplement for the growth phase (content per kg of diet): Vit. A, 2,148 UI; Vit. D_3_, 1,225 UI; Vit. E, 3,100 UI; Vit. K_3_, 1.5 mg; Vit. B_1_, 1.6 mg; Vit. B_12_, 16.7 μg; Riboflavin, 5.3 mg; Pyridoxine, 2.5 mg; Niacin, 36 mg; Pantothenic acid, 13 mg; Folic acid, 0.8 mg; *D*-biotin, 0.1 mg; choline chloride, 270; BHT, 5.8; Iron, 50 mg; Copper, 12 mg; Iodine, 0.9 mg; Zinc, 50 mg; Manganese, 60 mg; Selenium, 0.2 mg; Cobalt, 0.2 mg.

2) Kaolin – The addition of supplemental Gly and L-Thr were provided to the basal diet at the expense of the kaolin to derive the dietary treatments.

3) The analyzed values for total amino acids are in parentheses.

**Table 2:** Performance of broilers fed diets with reduced protein and levels of glycine equivalent (Gly_equi_) and standardized ileal digestible threonine (SID Thr) from days 21 to 42^1)^

Gly_equi_ (%)	SID Thr (%)	Weight gain (g)	Feed intake (g)	Feed:gain (g/g)
1.16	0.58	1,905^b^	3,326	1.75
1.26	0.58	1,927^b^	3,333	1.73
1.36	0.58	1,972^a^	3,371	1.71
1.46	0.58	1,987^a^	3,377	1.70
1.56	0.58	1,977^a^	3,424	1.73
1.16	0.68	1,903^b^	3,212	1.69
1.26	0.68	2,022^a^	3,471	1.72
1.36	0.68	2,020^a^	3,402	1.69
1.46	0.68	2,018^a^	3,367	1.67
1.56	0.68	1,988^ab^	3,408	1.72
1.16	0.78	1,987^a^	3,352	1.69
1.26	0.78	1,930^b^	3,362	1.75
1.36	0.78	1,928^b^	3,342	1.73
1.46	0.78	1,921^b^	3,381	1.76
1.56	0.78	1,924^b^	3,318	1.73
SEM	32.84	62.64	0.02	
Gly_equi_ (%)				
1.16		1,932	3,297	1.71
1.26		1,959	3,389	1.73
1.36		1,974	3,371	1.71
1.46		1,975	3,375	1.71
1.56		1,963	3,383	1.72
SEM		18.96	36.17	0.01
SID Thr (%)				
0.58		1,953^ab^	3,366	1.72^ab^
0.68		1,990^a^	3,372	1.70^b^
0.78		1,938^b^	3,351	1.73^a^
SEM		14.69	28.01	0.01
ANOVA		p-values		
Gly_equi_		0.19	0.17	0.76
SID Thr		0.04	0.86	0.03
Glye_qui_×SID Thr		0.04	0.43	0.34

SEM, standard error of the mean; ANOVA, analysis of variance.

1) Data represent the means of 5 replicate pens per treatment (treatments = 15; n = 5).

a,b Means in columns followed by different superscript are statistically different (p<0.05).

**Table 3:** Creatine content in pectoral muscle and relative weight of liver and pancreas of broilers fed diets with reduced protein and levels of glycine equivalent (Gly_equi_) and standardized ileal digestible threonine (SID Thr) at 421) days of age

Gly_equi_ (%)	SID Thr (%)	Pectoral pectoral muscular creatine (mg/g)	Liver (%)	Pancreas (%)
1.16	0.58	3.84^b^	2.70	0.25
1.26	0.58	4.15^ab^	2.58	0.26
1.36	0.58	4.72^a^	2.64	0.26
1.46	0.58	4.22^ab^	2.48	0.26
1.56	0.58	4.19^b^	2.44	0.23
1.16	0.68	4.31^ab^	2.54	0.28
1.26	0.68	3.94^a^	2.33	0.24
1.36	0.68	4.86^a^	2.80	0.27
1.46	0.68	5.31^a^	2.53	0.26
1.56	0.68	4.85^a^	2.60	0.27
1.16	0.78	3.33^b^	2.49	0.26
1.26	0.78	4.52^a^	2.40	0.23
1.36	0.78	5.00^a^	2.64	0.27
1.46	0.78	5.24^a^	2.59	0.25
1.56	0.78	5.26^a^	2.41	0.24
SEM		0.32	0.14	0.02
Gly_equi_ (%)				
1.16		3.83	2.58	0.26
1.26		4.20	2.44	0.24
1.36		4.86	2.69	0.27
1.46		4.92	2.53	0.26
1.56		4.77	2.48	0.25
SEM		0.20	0.09	0.01
SID Thr (%)				
0.58		4.23^b^	2.57	0.25
0.68		4.66^a^	2.56	0.26
0.78		4.67^a^	2.51	0.25
SEM		0.15	0.09	0.01
ANOVA		p-values		
Gly_equi_		Q^2)^ (0.008)	0.50	0.62
SID Thr		0.048	0.29	0.85
Gly_equi_×Thr		0.033	0.30	0.80

SEM, standard error of the mean; ANOVA, analysis of variance; Q, quadratic effect.

1) Data represent the means of 5 replicate pens per treatment (treatments = 15; n = 5).

2) Y= –20.714+34.851*x*–11.857*x*^2^ (*R*^2^ = 0.95); maximum point = 1.47% Gly_equi_.

a,b Means in columns followed by different superscript letters are statistically different (p<0.05).

**Table 4:** Carcass yield and cut parts of broilers fed diets with reduced protein and levels of glycine equivalent (Gly_equi_) and standardized ileal digestible threonine (SID Thr) at 42^1)^ days of age

Gly_equi_ (%)	SID Thr (%)	Carcass yield (%)	Breast yield (%)	Thigh+Drumstick (%)	Abdominal fat (%)
1.16	0.58	70.41^b^	40.72	34.25	2.80
1.26	0.58	70.94^ab^	41.74	34.10	2.71
1.36	0.58	71.50^ab^	41.07	35.15	2.60
1.46	0.58	71.72^ab^	42.09	35.14	2.31
1.56	0.58	72.50^a^	42.57	34.11	2.28
1.16	0.68	70.79^b^	41.4	34.07	3.31
1.26	0.68	71.66^ab^	43.89	33.81	2.35
1.36	0.68	73.03^a^	43.69	34.30	2.28
1.46	0.68	71.80^ab^	42.46	33.79	2.20
1.56	0.68	71.44^ab^	40.89	35.48	1.96
1.16	0.78	72.23^a^	42.54	34.61	2.52
1.26	0.78	72.01^a^	41.69	34.82	2.71
1.36	0.78	71.25^ab^	43.23	34.77	2.00
1.46	0.78	70.61^ab^	41.54	34.66	2.31
1.56	0.78	70.09^b^	40.97	34.95	2.58
SEM	0.67	0.76	0.54	0.27	
Gly_equi_ (%)					
1.16		71.14	41.55	34.31	2.88
1.26		71.54	42.44	34.24	2.59
1.36		71.93	42.66	34.74	2.29
1.46		71.37	42.03	34.53	2.27
1.56		71.34	41.48	34.85	2.27
SEM		0.39	0.44	0.31	0.16
SID Thr (%)					
058		71.41	41.64^b^	34.55	2.54
0.68		71.74	42.47^a^	34.29	2.42
0.78		71.24	41.99^ab^	34.76	2.42
SEM		0.30	0.34	0.24	0.12
ANOVA		p-values			
Gly_equi_		0.82	Q^2)^ (0.03)	0.99	L^3)^ (0.01)
SID Thr		0.44	0.05	0.53	0.70
Gly_equi_×SID Thr		0.002	0.08	0.59	0.10

SEM, standard error of the mean; ANOVA, analysis of variance; Q, quadratic effect; L, linear effect.

1) Data represent the means of 5 replicate pens per treatment (treatments = 15; n = 5)

2) Y = 6.0341+72.035*x*–26.69*x*^2^ (*R*^2^ = 0.93); maximum point = 1.35% Gly_equi_.

3) Y = 4.5331 − 1.5233*x* (R^2^ = 0.81).

a,b Means with different letters in the same column differ significantly (p<0.05).

**Table 5:** Blood biochemical indices^1)^ of broilers fed diets with reduced protein and levels of glycine equivalent (Gly_equi_) and standardized ileal digestible threonine (SID Thr) at 42 days of age

Gly_equi_ (%)	SID Thr (%)	Triglycerides (mg/dL)	Uric acid (mg/dL)	Albumin (g/dL)	Total protein (g/dL)	Creatine (mg/dL)	Glucose (mg/dL)	Ammonia (mg/dL)
1.16	0.58	68.21	4.78ª	1.60	1.67	0.22	265.09	3.27
1.26	0.58	84.50	4.65ª	1.53	1.74	0.22	303.73	2.86
1.36	0.58	79.32	3.00^b^	1.62	1.96	0.25	262.02	3.48
1.46	0.58	64.83	2.47^b^	1.48	1.83	0.21	267.84	3.12
1.56	0.58	57.8	2.25^b^	1.52	1.75	0.23	242.6	3.05
1.16	0.68	58.10	2.91ª	1.47	1.79	0.24	245.91	3.20
1.26	0.68	75.94	2.54^b^	1.51	1.83	0.23	257.99	3.01
1.36	0.68	53.93	3.09ª	1.57	1.84	0.26	261.47	4.13
1.46	0.68	47.33	2.49^b^	1.54	1.93	0.23	252.46	4.45
1.56	0.68	67.72	3.88ª	1.62	1.92	0.23	256.32	3.51
1.16	0.78	57.38	2.75ª	1.52	1.82	0.27	257.34	3.08
1.26	0.78	64.74	3.31ª	1.58	1.82	0.23	256.79	3.57
1.36	0.78	65.52	3.27ª	1.73	2.05	0.25	257.29	3.47
1.46	0.78	60.87	3.03ª	1.42	1.92	0.26	252.40	3.19
1.56	0.78	56.96	2.78ª	1.54	1.80	0.25	250.30	3.65
SEM		13.25	0.58	0.09	0.08	0.02	15.31	0.46
Gly_equi_ (%)								
1.16		61.23	3.48	1.53	1.76	0.25	256.11	3.19
1.26		75.06	3.50	1.54	1.80	0.23	272.84	3.15
1.36		66.25	3.12	1.64	1.95	0.25	260.26	3.69
1.46		57.67	2.67	1.48	1.89	0.23	257.57	3.59
1.56		60.83	2.97	1.56	1.82	0.24	249.74	3.40
SEM		7.50	0.33	0.05	0.05	0.01	8.84	0.32
SID Thr (%)								
0.58		70.93	3.43	1.55	1.79	0.23	268.26	3.16
0.68		60.6	2.98	1.54	1.86	0.24	254.83	3.66
0.78		61.09	3.03	1.56	1.88	0.25	254.82	3.39
SEM		5.73	0.26	0.04	0.04	0.01	6.85	0.26
ANOVA		p-values						
Gly_equi_		0.29	0.08	0.95	Q^2)^ (0.04)	0.48	0.31	0.25
SID Thr		0.17	0.35	0.96	0.22	0.12	0.27	0.16
Gly_equi_×SID Thr		0.87	0.002	0.38	0.68	0.14	0.35	0.80

SEM, standard error of the mean; ANOVA, analysis of variance; Q, quadratic effect.

1) Data represent the means of 5 replicate pens per treatment (treatments = 15; n = 5).

2) Y= −3,991+8.448*x*–3.0238*x*^2^ (*R*^2^ = 0.76); maximum point = 1.40% Gly_equi_,

a,b Means with different letters in the same column differ significantly (p<0.05).

**Table 6:** Thiobarbituric acid reactive substances (TBARS) values^1)^ expressed in mg of malonaldehyde (MDA/kg) of breast meat from broilers fed diets with reduced protein and levels of glycine equivalent (Gly_equi_) and standardized ileal digestible threonine at 42 days of age

Gly_equi_ (%)	SID Thr (%)	MDA/kg (mg)
1.16	0.58	0.70
1.36	0.58	0.87
1.56	0.58	0.55
1.16	0.68	0.82
1.36	0.68	0.40
1.56	0.68	0.58
1.16	0.78	0.71
1.36	0.78	0.78
1.56	0.78	0.48
SEM	0.09	
Gly_equi_ (%)		
1.16		0.74^a^
1.36		0.68^a^
1.56		0.54^b^
SEM		0.05
SID Thr (%)		
0.58		0.71
0.68		0.60
0.78		0.66
SEM		0.05
ANOVA		p-values
Gly_equi_		0.03
SID Thr		0.81
Gly_equi_×SID Thr		0.05

SID Thr, standardized ileal digestible threonine; SEM, standard error of the mean; ANOVA, analysis of variance.

1) Values represent the means of 5 replicate pens per treatment (treatments = 9; n = 3).

a,b Means with different letters in the same column differ significantly (p<0.05).

## References

[1] Hilliar M, Hargreave G, Girish CK, Barekatain R, Wu SB, Swick RA (2020). Using crystalline amino acids to supplement broiler chicken requirements in reduced protein diets. Poult Sci.

[2] Liu SY, Macelline SP, Chrystal PV, Selle PH (2021). Progress towards reduced-crude protein diets for broiler chickens and sustainable chicken meat production. J Anim Sci Biotechnol.

[3] Hejdysz M, Bogucka J, Ziółkowska E (2022). Effects of low crude protein content and glycine supplementation on broiler chicken performance, carcass traits, and litter quality. Livest Sci.

[4] Dean DW, Bidner TD, Southern LL (2006). Glycine supplementation to low protein, amino acid-supplemented diets supports optimal performance of broiler chicks. Poult Sci.

[5] Saleh AA, Amber KA, Soliman MM (2021). Effect of low protein diets with amino acids supplementation on growth performance, carcass traits, blood parameters and muscle amino acids profile in broiler chickens under high ambient temperature. Agriculture.

[6] Ospina-Rojas IC, Murakami AE, Duarte CRA, Eyng C, Oliveira CAL, Janeiro V (2014). Valine, isoleucine, arginine and glycine supplementation of low-protein diets for broiler chickens during the starter and grower phases. Br Poult Sci.

[7] Siegert W, Wild KJ, Schollenberger M, Helmbrecht A, Rodehutscord M (2016). Effect of glycine supplementation in low protein diets with amino acids from soy protein isolate or free amino acids on broiler growth and nitrogen utilization. Br Poult Sci.

[8] Ospina-Rojas IC, Murakami AE, Moreira I, Picoli KP, Rodrigueiro RJB, Furlan AC (2013). Dietary glycine+serine responses of male broilers given low-protein diets with different concentrations of threonine. Br Poult Sci.

[9] Baker DH, Hill TM, Kleiss AJ (1972). Nutritional evidence concerning formation of glycine from threonine in the chick. J Anim Sci.

[10] Ospina-Rojas IC, Murakami AE, Oliveira CAL, Guerra AFQG (2013). Supplemental glycine and threonine effects on performance, intestinal mucosa development, and nutrient utilization of growing broiler chickens. Poult Sci.

[11] Hilliar M, Huyen N, Girish CK, Barekatain R, Wu S, Swick RA (2019). Supplementing glycine, serine, and threonine in low protein diets for meat type chickens. Poult Sci.

[12] Davis AJ, Austic RE (1994). Dietary threonine imbalance alters threonine dehydrogenase activity in isolated hepatic mitochondria of chicks and rats. J Nutr.

[13] Corzo A, Kidd MT, Dozier WA, Kerr BJ (2009). Dietary glycine and threonine interactive effects in broilers. J Appl Poult Res.

[14] Siegert W, Ahmadi H, Helmbrecht A, Rodehutscord M (2015). A quantitative study of the interactive effects of glycine and serine with threonine and choline on growth performance in broilers. Poult Sci.

[15] Aguihe PC, Hirata KA, Ospina-Rojas CI (2022). Effect of glycine equivalent levels in low protein diet containing different SID threonine concentrations on performance, serum metabolites and pectoral muscular creatine of broiler chickens. Ital J Anim Sci.

[16] Rostagno HS, Albino LFT, Donzele JL, Viçosa MG (2011). Brazilian tables for poultry and swine: feed composition and nutritional requirements.

[17] Hofmann P, Siegert W, Kenéz Á, Naranjo VD, Rodehutscord M (2019). Very low crude protein and varying glycine concentrations in the diet affect growth performance, characteristics of nitrogen excretion, and the blood metabolome of broiler chickens. J Nutr.

[18] Elahi U, Wang J, Ma YB, Wu SG, Qi GH, Zhang HJ (2020). The response of broiler chickens to dietary soybean meal reduction with glycine and cysteine inclusion at marginal sulfur amino acids (SAA) deficiency. Animals.

[19] Tietz NW (1986). Fundamentals of clinical chemistry.

[20] Ishihara A, Kurahasi K, Uehara H (1972). Enzymatic determination of ammonia in blood and plasma. Clin Chim Acta.

[21] Chamruspollert M, Pesti GM, Bakalli RI (2002). Dietary interrelationships among arginine, methionine, and lysine in young broiler chicks. Br J Nutr.

[22] Sørensen G, Jørgensen SS (1996). A critical examination of some experimental variables in the 2-thiobarbituric acid (TBA) test for lipid oxidation in meat products. Eur Food Res Technol.

[23] Kriseldi R, Tillman PB, Jiang Z, Dozier WA (2018). Effects of feeding reduced crude protein diets on growth performance, nitrogen excretion, and plasma uric acid concentration of broiler chicks during the starter period. Poult Sci.

[24] Greenhalgh S, McInerney BV, McQuade LR (2020). Capping dietary starch:protein ratios in moderately reduced crude protein, wheat-based diets showed promise but further reductions generated inferior growth performance in broiler chickens. Anim Nutr.

[25] Chrystal PV, Greenhalgh S, Selle PH, Liu SY (2020). Facilitating the acceptance of tangibly reduced-crude protein diets for chicken-meat production. Anim Nutr.

[26] Cobb-Vantress Inc (c2008). Broiler performance and nutrition supplement 48 [Internet].

[27] Law FL, Zulkifli I, Soleimani AF, Liang JB, Awad EA (2018). The effects of low protein diets and protease supplementation on broiler chickens in a hot and humid tropical environment. Asian-Australas J Anim Sci.

[28] Chrystal PV, Moss AF, Yin D (2020). Glycine equivalent and threonine inclusions in reduced-crude protein, maize-based diets impact on growth performance, fat deposition, starch-protein digestive dynamics and amino acid metabolism in broiler chickens. Anim Feed Sci Technol.

[29] Bernardino VMP, Albino LFT, Rostagno HS (2011). Effect of different digestible threonine: digestible lysine ratios, with or without glycine supplementation, on the enzymatic activity in broiler chicks. R Bras Zootec.

[30] Min YN, Liu SG, Qu ZX, Meng GH, Gao YP (2017). Effects of dietary threonine levels on growth performance, serum biochemical indexes, antioxidant capacities, and gut morphology in broiler chickens. Poult Sci.

[31] Najafi R, Ahmar R, Tazehkand G (2017). Effect of different dietary threonine levels on optimal growth performance and intestinal morphology in 1–14 days old Ross 308 broilers. Rev Bras Cienc Avic.

[32] Silva CA, Cancelliero KM (2006). Effect of oral creatine supplementation in skeletal muscle of rat immobilized hindlimb. Rev Bras Nutr Clin.

[33] Lemme A, Ringel J, Rostagno HS, Redshaw MS, 16th European Symposium on Poultry Nutrition (2007). Supplemental guanidino acetic acid improved feed conversion, weight gain, and breast meat yield in male and female broilers.

[34] Tossenberger J, Rademacher M, Németh K, Halas V, Lemme A (2016). Digestibility and metabolism of dietary guanidino acetic acid fed to broilers. Poult Sci.

[35] Kidd MT, Maynard CW, Mullenix GJ (2021). Progress of amino acid nutrition for diet protein reduction in poultry. J Anim Sci Biotechnol.

[36] Majdeddin M, Braun U, Lemme A (2020). Guanidinoacetic acid supplementation improves feed conversion in broilers subjected to heat stress associated with pectoral muscular creatine loading and arginine sparing. Poult Sci.

[37] Burke DG, Candow DG, Chilibeck PD (2008). Effect of creatine supplementation and resistance-exercise training on muscle insulin-like growth factor in young adults. Int J Sport Nutr Exerc Metab.

[38] Namroud NF, Shivazad M, Zaghari M, Shahneh AZ (2010). Effects of Glycine and glutamic acid supplementation to low protein diets on performance, thyroid function and fat deposition in chickens. South Afr J Anim Sci.

[39] Akinde DO (2014). Amino acid efficiency with dietary glycine supplementation: part 1. Worlds Poult Sci J.

[40] Wang WW, Wang J, Wu SG, Zhang HJ, Qi GH (2020). Response of broilers to gradual dietary protein reduction with or without an adequate glycine plus serine level. Ital J Anim Sci.

[41] McCarty MF, O’Keefe JH, DiNicolantonio JJ (2018). Dietary glycine is rate-limiting for glutathione synthesis and may have broad potential for health protection. Ochsner J.

[42] Surai PF, Kochish II, Fisinin VI, Kidd MT (2019). Antioxidant defence systems and oxidative stress in poultry biology: an update. Antioxidants.

[43] Ponnampalam EN, Kiani A, Santhiravel S, Holman BWB, Lauridsen C, Dunshea FR (2022). The importance of dietary antioxidants on oxidative stress, meat and milk production, and their preservative aspects in farm animals: antioxidant action, animal health, and product quality. Animals.

[44] Senthilkumar R, Sengottuvelan M, Nalini N (2003). Protective effect of glycine supplementation on the levels of lipid peroxidation and antioxidant enzymes in the erythrocytes of rats with alcohol induced liver injury. Cell Biochem Funct.

[45] Gao L, Zhang C, Zheng Y (2023). Glycine regulates lipid peroxidation promoting porcine oocyte maturation and early embryonic development. J Anim Sci.

[46] Deng C, Zheng J, Zhou H, You J, Li G (2023). Dietary glycine supplementation prevents heat stress-induced impairment of antioxidant status and intestinal barrier function in broilers. Poult Sci.

